# Surface-Driven Phase Segregation in Conducting Polymer Thin Films Enables High Selectivity and Storage Stability of Chemiresistive Sensors in Humid Air

**DOI:** 10.3390/polym17070979

**Published:** 2025-04-03

**Authors:** Jianan Weng, Wei Wu, Minghao Qian, Jiarui Zhang, Shuhua Zhang, Zhi Geng, Bo Zhu

**Affiliations:** 1 School of Materials Science and Engineering, Shanghai University, 99 Shangda Road, Baoshan, Shanghai 200444, China; wengjn@foxmail.com (J.W.); wu10001300@163.com (W.W.); mhqian2001@163.com (M.Q.); zjr1178713489@gmail.com (J.Z.); shzhang1992@shu.edu.cn (S.Z.); gengzhi@shu.edu.cn (Z.G.); 2 Shanghai Engineering Research Center of Organ Repair, 99 Shangda Road, Baoshan, Shanghai 200444, China; 3 Joint International Research Laboratory of Biomaterials and Biotechnology in Organ Repair (Ministry of Education), 99 Shangda Road, Baoshan, Shanghai 200444, China

**Keywords:** chemiresistive sensor, organophosphate, conductive copolymer, 3,4-ethylenedioxythiophene, sensing selectivity, water vapor resistance

## Abstract

Chemiresistive sensors integrated with functionalized conductive polymers have emerged as promising candidates for wearable applications, offering adequate protection against highly toxic and widely prevalent organophosphate compounds, due to their high sensitivity, room-temperature operation, and straightforward fabrication process. However, these chemiresistive sensors exhibit poor resistance to water vapor due to the intrinsic properties of these conducting polymers, likely leading to false sensor alarms. In this study, we engineered a series of water-vapor-resistant, yet organophosphate-sensitive, conducting polymers by electro-copolymerizing hexafluoroisopropanol (HFIP)-grafted 3,4-ethylenedioxythiophene (EDOT-HFIP) with EDOT comonomers bearing hydrophobic alkyl groups of varying lengths (ethyl, butyl, and hexyl). The typical results indicated that increasing the alkyl length and alkyl-bearing EDOT comonomer composition significantly enhanced the water resistance of the EDOT-HFIP copolymers and the copolymer-integrated chemiresistive sensor, but this improvement came at the unacceptable cost of compromising the organophosphate sensitivity. To address this issue, we developed a surface-driven phase-segregation strategy to enrich the alkyl chains on the surface while concentrating the HFIP groups beneath it by treating the silica substrates using oxygen plasma before polymer spin coating, thus decoupling and optimizing the two mutually competing characteristics. Finally, the chemiresistive sensor integrated with the EDOT-HFIP copolymer containing 10% hexyl-grafted EDOT comonomer exhibited an organophosphate (DMMP) resistive response 657 times higher than that to water vapor, and more than two times that of a PEDOT-HFIP sensor, while preserving the original specific sensitivity of the PEDOT-HFIP sensor. Furthermore, it demonstrated a markedly improved shelf storage stability, being directly exposed to air for 14 days without any special protection. We envision that this surface-driven phase-segregation strategy could offer a promising solution to the significant challenge of air moisture interference in highly sensitive polymer sensors, promoting their practical use in real-world applications.

## 1. Introduction

Organophosphate compounds are used in agriculture as pesticides to control or eliminate plant and animal pests (pathogenic fungi, insects, rodents, and weeds). Most organophosphate compounds can cause various neurological diseases at concentrations far below human visual and olfactory threshold levels, posing a serious threat to human health. There is an urgent need for a miniaturized sensor capable of sensitively monitoring organophosphate compounds to protect individuals who may be exposed to environments contaminated with these toxic substances [1]. Chemoresistive sensors, which work by detecting the resistance changes at contact with a target gas, are considered an attractive option for this wearable application, due to their simple device structure and circuit integration, small size, low-cost manufacturing, and low power consumption [2,3].

The sensing materials are a crucial component of chemical gas sensors, as they are one of the most critical factors determining a sensor’s sensitivity, selectivity, and stability [3]. Metal oxide materials, represented by SnO_2_ [4,5,6], WO₃ [7], and ZnO [8], were initially applied for organophosphorus sensing due to their stability advantages. However, they suffer from high operating temperatures (above 200 °C), high power consumption, and poor selectivity. Carbon nanomaterials, such as graphene [9,10,11,12,13,14] and carbon nanotubes [15,16,17,18,19], capable of room-temperature sensing, have recently been considered an alternative to metal oxides. Carbon nanomaterials typically require surface modification [9,11,12] or compositing with other sensing materials [13,14,18,19] to be used for organophosphate sensing due to their intrinsic lack of specific adsorption capabilities. Despite their high specific surface area, which enhances their sensitivity, their high surface energy typically facilitates their aggregation during storage, leading to poor stability [20]. Functionalized conductive polymers have emerged as ideal materials for organophosphate sensing due to their high sensitivity at room temperature, which results from the sensitive doping/dedoping process [21,22], easy solution processing [22], and potential storage stability attributed to their intrinsic homogeneity. Our previous study revealed that a chemiresistive sensor integrated with a hexafluoroisopropanol (HFIP)-grafted 3,4-ethylenedioxythiophene homopolymer (PEDOT-HFIP) can detect dimethyl methylphosphonate (DMMP) at 10 ppb [23]. The trace detection capability of a PEDOT-HFIP sensor arises from the strong specific interaction between the HFIP groups and organophosphate compounds, leading to the dedoping of PEDOT-HFIP.

However, water molecules in the air can interfere with the specific sensing of chemiresistive sensors, [24] whether based on carbon nanomaterials or functionalized conducting polymers, as these materials inherently absorb or interact with water molecules through their skeleton structures or polar functionalities. This persistent moisture interference remains a significant challenge for the real-world application of chemiresistive sensors for either rapid detection or continuous monitoring scenarios. The existing solutions for these selectivity issues have predominantly relied on sensor array systems [25,26,27] that employ an orthogonal analysis of multi-sensor data. This method not only increases the complexity but also significantly raises the manufacturing costs. Consequently, developing intrinsically water-resistant sensing materials for chemiresistive sensors is highly desirable, as it would enable highly sensitive and selective organophosphate detection through simple and cost-effective methods.

In this study, we engineered a series of water-vapor-resistant, yet organophosphate-sensitive, conducting copolymers by partially substituting the side groups of a hexafluoroisopropanol (HFIP)-grafted 3,4-ethylenedioxythiophene (EDOT-HFIP) homopolymer, a high-performance organophosphate sensing material, with alkyl functionalities of varying lengths (ethyl, butyl, and hexyl groups). This modification was achieved by quantitatively copolymerizing the EDOT-HFIP with hydrophobicity-tailored EDOT comonomers bearing alkyl groups of varying lengths (EDOT-C_2_/C_4_/C_6_), thereby precisely modulating the polymer’s water vapor resistance with the organophosphate sensitivity. These functionalized EDOT copolymer thin films were spin-coated onto interdigitated electrodes to fabricate water-vapor-resistant chemiresistive sensors for detecting organophosphate (Figure S1). The typical results indicated that increasing the alkyl group substitution or the alkyl group length increased the water vapor resistance of the chemiresistive sensors, but came at the expense of reducing their specific sensitivity, likely due to the dilution of the HFIP groups. Fortunately, pretreating the silica substrate using oxygen plasma vastly enriched the alkyl chains on the surface while concentrating the HFIP groups beneath it, thus decoupling and optimizing the two mutually competing characteristics. As a result, the chemiresistive sensor integrated with the EDOT-HFIP copolymer containing 10% hexyl-grafted EDOT comonomers achieved an organophosphate (DMMP) resistive response 657 times higher than that to water vapor, and more than twice that of a PEDOT-HFIP sensor, while maintaining the original specific sensitivity of the PEDOT-HFIP sensor. Finally, it demonstrated a markedly improved shelf storage stability after being directly exposed to air without any special protection for 14 days. We envision that this surface treatment strategy to enhance phase segregation could provide a viable solution for the air moisture interference issue of thin-film sensors, and thus promote their practical application in the real world.

## 2. Experimental Section

### 2.1. Materials

1,1,1-Trifluoro-2-(Trifluoromethyl)-4-Penten-2-ol (purified ≥ 98.0%, CAS: 646-97-9) and 1,4,7,10,13,16-Hexaoxacyclooctadecane (18-crown-6, purified ≥ 98%, CAS: 17455-13-9) were purchased from Tokyo Chemical Industry (TCI) (Tokyo, Japan). 3,4-Dimethoxythiophene (98%+, CAS: 51792-34-8), Tert-butyl alcohol (purified ≥ 99.5%, CAS: 25725-11-5), Potassium permanganate (purified ≥ 99.5%, CAS: 7722-64-7), P-tolunesulfonic acid monohydrate (purified ≥ 98%, CAS: 104-15-4), 2’-hydroxymethyl-3,4-ethylenedioxythiophene (EDOT-OH, purified ≥ 99%, CAS: 146796-02-3), Sodium Hydride (NaH, purified ≥ 60%, CAS: 7646-69-7), Bromoethane (purified ≥ 99%, CAS: 74-96-4), Butyl Bromide (purified ≥ 99%, CAS: 109-65-9), Hexyl Bromide (purified ≥ 99%, CAS: 111-25-1), Lithium chloride (purified ≥ 99.0%, CAS: 7447-41-8), Sodium dodecyl sulfate (purified ≥ 99.0%, CAS: 151-21-3), Dimethyl methylphosphonate (DMMP, purified ≥ 98.0%, CAS: 756-79-6), ethyl acetate (purified ≥ 99.5%, CAS: 141-78-6), magnesium sulfate anhydrous (purified ≥ 98%, CAS: 7487-88-9), hexane (purified ≥ 99.5%, CAS: 92112-69-1), sodium hydrogen carbonate (purified ≥ 99.5%+, CAS: 144-55-8), and isopropyl alcohol (purified ≥ 99%, CAS: 67-63-0) were purchased from Shanghai Titan Scientific Co., Ltd. (Shanghai, China). Chloroform (purified ≥ 99.5%+, CAS: 67-66-3) and acetone (purified ≥ 99.5%+, CAS: 67-64-1) were purchased from Sinopharm Chemical Reagent Co., Ltd. (Shanghai, China).

### 2.2. Synthesis of EDOT-HFIP and EDOT-C_n_

**Figure 1:**
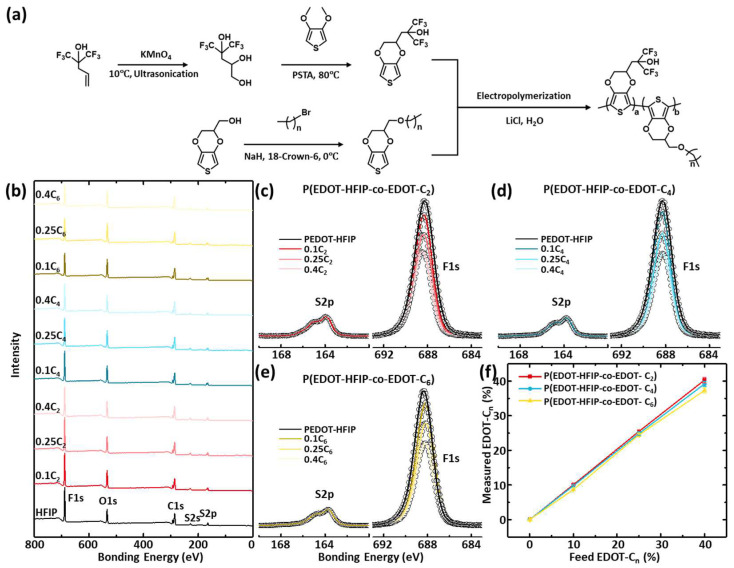
(**a**) Schematic presentation of chemical pathway of EDOT-HFIP monomer, EDOT-Cn monomer, and P(EDOT-HFIP-co-EDOT-Cn). (**b**–**e**) XPS survey spectra and high-resolution S2p and F1s spectra of P(EDOT-HFIP-co-EDOT-C2)s, P(EDOT-HFIP-co-EDOT-C4)s, and P(EDOT-HFIP-co-EDOT-C6)s prepared with EDOT-C2/C4/C6 feed ratios of 0, 0.1, 0.25, and 0.4. These copolymer films were dip-coated onto pristine silica substrate. (**f**) Plots of EDOT-Cn compositions of P(EDOT-HFIP-co-EDOT-C2)s, P(EDOT-HFIP-co-EDOT-C4)s, and P(EDOT-HFIP-co-EDOT-C6)s, estimated from high-resolution spectral data (**c**–**e**).

Hexafluoroisopropyl alcohol-functionalized EDOT (EDOT-HFIP) was synthesized (Figure 1a) according to previously reported methods [23]. Briefly, a dry Schlenk flask was charged with 1,1,1-trifluoro-2-(trifluoromethyl)-4-penten-2-ol, potassium permanganate, and tert-butyl alcohol, followed by adding Milli-Q water. The flask was placed in a 15 °C water bath for 30 min. The reaction was quenched with sodium thiosulfate, and the mixture was extracted with ethyl acetate. The organic layers were washed with brine, dried (MgSO_4_), and concentrated. Purification by silica gel chromatography (EtOAc/hexane) yielded 3-hexafluoroisopropanol-1,2-diol as a white solid. P-Toluenesulfonic acid monohydrate, 3-hexafluoro-isopropanol-1,2-diol, and 3,4-dimethoxythiophene were added into a dried three-neck flask with stirring and nitrogen purge. Anhydrous toluene was added, and the mixture was heated to 90 °C in a thermostated bath for 4 h; the mixture was partitioned between water and ethyl acetate, and the organic layers were washed with NaHCO_3_ solution, dried (MgSO_4_), and concentrated. Silica gel chromatography (acetone/hexane) purification yielded EDOT-HFIP (42%) as a white solid. ^1^H NMR (600 MHz, d6-DMSO) was as follows: δ 8.25 (s, 1H), 6.60 (dd, 2H, J = 3.62, 3.54 Hz), 4.46 (s, 1H), 4.26 (dd, 1H, J = 11.6, 2.4 Hz), 4.24 (s, 1H), 4.12 (dd, 1H, J = 11.6, 7.2 Hz), 4.24 (dd, 1H, J =2.41, 2.26 Hz), 3.99 (m, 1H), and 2.29–2.17 (m, 2H).

**Figure 2:**
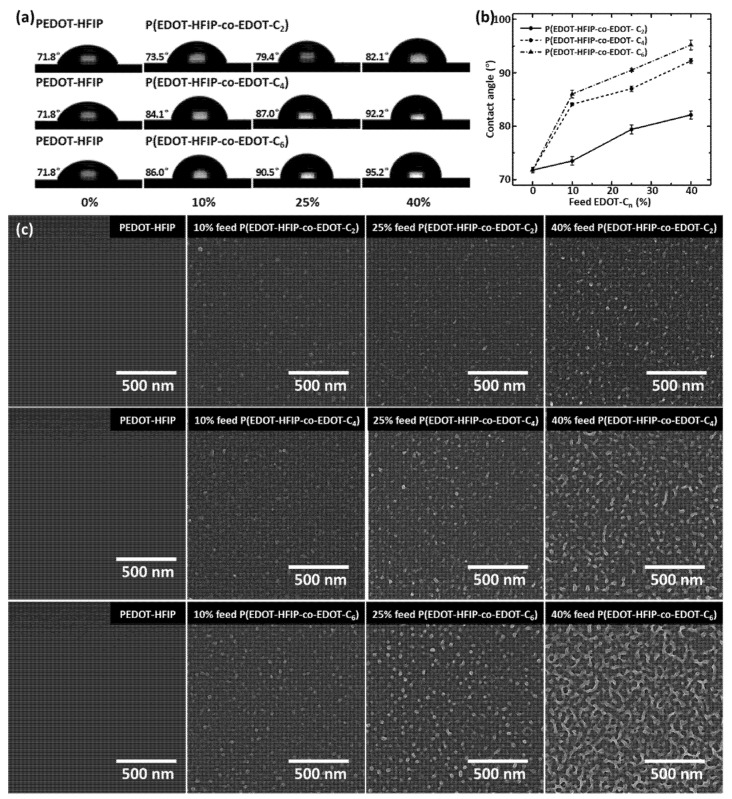
(**a**) Water static contact angles of PEDOT-HFIP and P(EDOT-HFIP-co-EDOT-Cn) thin films spin-coated onto plasma-treated silica substrates. (**b**) Plots of water static contact angles as function of EDOT-Cn feed ratio for P(EDOT-HFIP-co-EDOT-C2), P(EDOT-HFIP-co-EDOT-C4), and P(EDOT-HFIP-co-EDOT-C6) thin films. (**c**) SEM images of PEDOT-HFIP, P(EDOT-HFIP-co-EDOT-C2), P(EDOT-HFIP-co-EDOT-C4), and P(EDOT-HFIP-co-EDOT-C6) thin films.

Alkyl group-functionalized EDOT (EDOT-C_n_) monomers, where n indicates the carbon number of alkyl groups, were synthesized following the chemical pathway shown in Figure 2a, using EDOT-OH and a series of brominated alkanes as starting materials. A dry Schlenk flask was charged with EDOT-OH, NaH, 18-crown-6, and dry THF, and dried nitrogen was flowed through to remove water and oxygen. The flask was placed in a 0 °C water bath for 30 min. Bromoethane, butyl bromide, or hexyl bromide was slowly added, and the mixture was placed under nitrogen protection, in the dark, at 25 °C for 12 h. The reaction was quenched with water, and the mixture was extracted with ethyl acetate. The organic layers were washed with brine, dried (MgSO_4_), and concentrated. Purification by silica gel chromatography (ethyl acetate/hexane) yielded EDOT-C_2_ (98%) as a white solid, and EDOT-C_4_ (98%) and EDOT-C_6_ (97%) as pale-yellow viscous liquids. ^1^H NMR (600 MHz, Chloroform-d) of EDOT-C_2_ was as follows: δ 6.36 (d, J = 3.6 Hz, 2H), 4.33 (d, J = 5.8 Hz, 1H), 4.29–4.05 (m, 2H), 3.75–3.64 (m, 2H), 3.58 (d, J = 7.0 Hz, 2H), and 1.25 (t, J = 7.0 Hz, 3H). ^1^H NMR (600 MHz, Chloroform-d) of EDOT-C_4_ was as follows: δ 6.34 (q, J = 3.7 Hz, 2H), 4.34–4.30 (m, 1H), 4.28–4.06 (m, 2H), 3.72–3.60 (m, 2H), 3.52 (t, J = 6.6 Hz, 2H), 1.62–1.57 (m, 2H), 1.42–1.37 (m, 2H), and 0.95 (t, J = 7.4 Hz, 3H). ^1^H NMR (600 MHz, Chloroform-d) of EDOT-C_6_ was as follows: δ 6.34 (q, J = 3.7 Hz, 2H), 4.32 (m, J = 7.4, 6.0, 5.0, 2.3 Hz, 1H), 4.28–4.06 (m, 2H), 3.72–3.61 (m, 2H), 3.51 (t, J = 6.7 Hz, 2H), 1.61 (d, J = 6.0 Hz, 2H), 1.37–1.30 (m, 6H), and 0.92 (d, J = 6.7 Hz, 3H).

### 2.3. Fabrication of the Chemiresistive Sensor

The chemiresistive sensor consisted of a P(EDOT-HFIP-co-EDOT-C_n_)-sensitive membrane material assembled on a specially interdigitated electrode (IDE), designed as shown in Figure S2. The process for IDE is shown in Figure S3, during which a SiO_2_ layer was first thermally oxidized as an insulating layer on a four-inch monocrystalline silicon wafer. Then, the electrode pattern was etched onto the insulating layer by photolithography, followed by thermal vapor deposition of chromium (Ge as the adhesive layer) and gold (Au as the conductive layer), respectively. Finally, the photoresist was removed to obtain a gold-forked finger electrode (photograph in Figure S4).

After being dissolved in acetone, the sensitive material was coated onto the IDE using spin coating. A spinning speed of 6000 rpm was applied to spin-coat the P(EDOT-HFIP-co-EDOT-C_n_) with a commercial spin coater (LEBO Science, EZ4). Once the spin-coating process was completed, the samples were dried in a vacuum at 25 °C for 4 h.

### 2.4. Materials and Interface Characterization

The elements of the sample were analyzed using an X-ray photoelectron spectrometer (XPS) (model: Thermo Fisher Scientific (Shanghai, China); excitation source: Al Kα X-ray; hv = 1486.6 eV; spot size: 400 μm; operating voltage: 12 kV; tungsten filament current: 6 mA; electron emission angle: 60°; full-scan pass energy: 150 eV and step size: 1 eV; narrow-scan pass energy: 50 eV and step size: 0.1 eV).

The water static contact angle of the P(EDOT-HFIP-co-EDOT-C_n_) polymer thin films was measured using a static contact angle measurement system (Theta Flex, Biolin Scientific, Gothenburg, Sweden) and recorded with a high-speed camera (T200/C) at ambient temperature (25 °C). A 4 μL drop of water was dispensed onto the surface of the films. Each sample was measured three times to obtain an average value.

The scanning electron microscope (SEM) views of polymer thin films were observed on a JSM-7500F (JEOL Japan Electronics Co., Ltd., Shanghai, China) (electron gun: Schottky field emission gun; resolution: 1.0 nm at 15 keV ~ 1.6 nm at 1 keV; accelerating voltage: 10 keV; probe current: 10 nA). After spin coating the P(EDOT-HFIP-co-EDOT-C_n_) copolymers onto the interdigitated electrode (IDE), it was fixed onto an SEM stage with carbon conductive tapes (NISSHIN EM) before measurement.

### 2.5. Evaluation of Chemiresistive Sensors

The chemiresistive sensors were placed in a homemade tetrafluoroethylene test chamber with fixtures for sensing tests. The concentrations of the various gases were controlled by an MF-3D liquid organic solvent dynamic gas distribution system (China National Metrology Technology Development Corporation, Beijing, China). Real-time resistance monitoring was performed at a constant current of 1 μA (denoted as ΔR/R_0_ = (R − R_0_)/R_0_, where R and R_0_ represent the real-time and initial resistance, respectively) using an Autolab electrochemical workstation (PGSTAT128N, Metrohm China Co., Ltd., Shanghai, China). Dimethyl methylphosphonate (DMMP) was chosen as the signature organophosphorus compound for testing, while nitrogen (N_2_) was used as the dilution gas to dilute DMMP and as a desorption gas to remove DMMP adsorbed onto the chemiresistive sensor.

### 2.6. Calculation of the Selectivity Coefficient

The selectivity coefficient (SC), calculated as the ratio of the sensor’s response to DMMP versus water vapor at equivalent concentrations, was determined using the following equation:

(1) $$SC=\frac{\Delta R/R_{0}(Target\;chemical,\;1\;ppm)}{\frac{\Delta R/R_{0}\left(Interfering\;chemical,\;200\;ppm\right)}{200}}$$

where $\Delta R/R_{0}(Target\;chemical,1\;ppm)$ is the response value of the sensor at 1 ppm of target chemical (DMMP), and $\Delta R/R_{0}(Interfering\;chemical,200\;ppm)$ is the response value of the sensor at 200 ppm of interfering chemical (water vapor).

## 3. Results and Discussion

### 3.1. Synthesis of P(EDOT-HFIP-Co-EDOT-C_n_)s

The EDOT-HFIP and EDOT monomers bearing alkyl groups of varying lengths (EDOT-C_n_) were synthesized following the chemical pathways presented in Figure 1a. The copolymers of the EDOT-HFIP with EDOT-C_2_, EDOT-C_4_, or EDOT-C_6_ with various compositions were electrodeposited onto Pt mesh by applying a constant potential of 1.13 v in an aqueous solution containing 20 mM of EDOT monomers with varying mole ratios of EDOT-C_n_ (0, 0.1, 0.25, and 0.4), 100 mM sodium dodecyl sulfate (SDS), and 300 mM of lithium chloride (LiCl), with a Ag/AgCl electrode as the reference (Figure S5I—V curves registered during the electrodepositions). These copolymers were collected by dissolving them in acetone and then purifying them through precipitation in chloroform.

The XPS survey spectra of the copolymers of the EDOT-HFIP and EDOT-C_n_ at various mole feed ratios are displayed in Figure 1b–f. All the copolymers exhibit elemental characteristic peaks identical to PEDOT-HFIP without discernible shifts in the corresponding bonding energies. This consistency across all the XPS spectra arises from the fact that copolymerizing EDOT-HFOP with EDOT-C_n_ does not introduce additional elements, therefore not significantly changing the chemical environment. The further analysis of the high-resolution XPS spectra (Figure 1c–e) was focused on the F1s peaks of the copolymers. For the convenience of the comparison, the intensities of the F1s peaks of all the copolymers were normalized by the intensity of the S2p peaks, as the S element is exclusively located in the backbone of all the EDOT monomers, making it appropriate as the internal reference. The results reveal that the intensities of the characteristic F1s peaks of the copolymers gradually decreased with increasing EDOT-C_n_ feed ratios. The actual compositions of these copolymers were calculated based on the intensities of the F1s and S2p peaks after considering the differences in their relative sensitivity factors. The dependences of the EDOT-C_n_ compositions of all the copolymers as a function of the feed ratios are plotted in Figure 1f. Clearly, the actual monomer composition of all the copolymers closely matches those in the feed, and are nearly independent of the alkyl group lengths of the comonomers, exhibiting a linear dependence on the monomer feed ratios. These results indicate that precise control over the copolymer composition can be achieved by adjusting the monomer ratios at the feed.

### 3.2. Surface-Driven Nano-Assembly and Phase Segregation

After carefully optimizing the conditions, we spin-coated 1.25 mg/mL of the copolymer solutions onto silica substrates that had been pretreated with oxygen plasma, forming copolymer thin films with a thickness of approximately 50 nm, ensuring compatibility with the dimensions of the interdigitated electrodes. These copolymer thin films’ water static contact angles were then measured to investigate the effects of the alkyl side group length and EDOT-C_n_ compositions on the surface properties. Figure 2a presents the water contact angles and corresponding water droplet images for all the copolymer thin films, with the PEDOT-HFIP polymer films as the control. As expected, the contact angles of the copolymer thin films increased with the EDOT-C_n_ compositions (Figure 2b) for all three copolymers, due to the intrinsically hydrophobic nature of the alkyl chains. Moreover, the incorporation of the butyl and hexyl groups led to a more pronounced increase in the water contact angle than the incorporation of the ethyl group, as the longer alkyl chains exhibited greater hydrophobicity [28]. Therefore, copolymerization with hydrophobic EDOT-C_n_s can effectively enhance the hydrophobicity of polymer films, highlighting their potential to improve the water vapor resistance of chemiresistive sensors.

We then used a scanning electron microscope (SEM) to investigate the surface morphologies of these P(EDOT-HFIP-co-EDOT-C_n_) copolymer films. As shown in Figure 2c, all the copolymer thin films presented with some nanorod structures, which were absent in the films coated on the untreated silica substrates (Figure S6). This suggests that the surface functionality activated by the oxygen plasma treatment, as revealed in our previous study [23], plays a crucial role in forming the nanostructures on the surface. Additionally, the nanorod-like structures were not observed on the surface of the EDOT-HFIP homopolymer thin films, whether coated on the plasma-treated or untreated silica surfaces [23]. This likely suggests that the presence of alkyl side groups is another critical factor driving the formation of nanostructures. Furthermore, the nanorod density increased with the EDOT-C_n_ composition and the alkyl group length, highlighting the significant role of the alkyl chain presence. Moreover, the nanorod diameter expanded from about 20 nm to approximately 50 nm when the alky group length was extended from ethyl to hexyl, further confirming the distinctive effect of the alkyl chain on the nanorod formation again.

**Figure 3:**
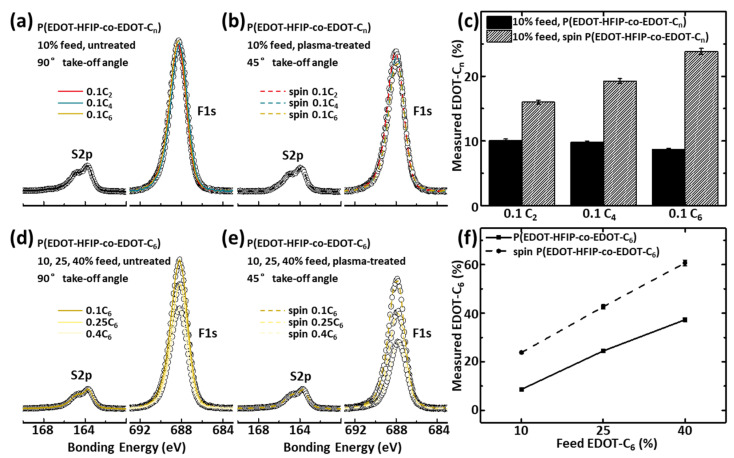
(**a**) Normalized high-resolution S2p and F1s spectra collected at 90° take-off angle for the P(EDOT-HFIP-co-0.1EDOT-Cn) synthesized under a 10% EDOT-Cn feed ratio and dip-coated on untreated silica substrates. (**b**) Normalized XPS high-resolution S2p and F1s spectra collected at 45° take-off angle for the P(EDOT-HFIP-co-0.1EDOT-Cn) copolymers synthesized under a 10% EDOT-Cn feed ratio and spin-coated on plasma-treated silica substrates. (**c**) Comparison of the EDOT-Cn compositions determined by the above XPS data between the P(EDOT-HFIP-co-0.1EDOT-Cn) films coated on the pristine silica substrate and those coated on the plasma-treated silica substrates. (**d**) Normalized high-resolution S2p and F1s spectra collected at 90° take-off angle for the P(EDOT-HFIP-co-EDOT-C6)s synthesized under 10%, 25%, and 40% EDOT-Cn feed ratios and dip-coated on untreated silica substrates. (**e**) Normalized high-resolution S2p and F1s spectra collected at 45° take-off angle for the P(EDOT-HFIP-co-EDOT-C6) synthesized under 10%, 25%, and 40% EDOT-Cn feed ratios and spin-coated on the plasma-treated silica substrates. (**f**) A comparison of the compositions, as determined by the XPS data, for the P(EDOT-HFIP-co-EDOT-C6) synthesized with EDOT-Cn feed ratios of 10%, 25%, and 40% and dip-coated on pristine silica substrates and spin-coated on plasma-treated silica substrates.

To further investigate the composition of the nanostructured surface and the driving forces behind the nano-assembly, we performed XPS measurements at a small take-off angle of 45° to analyze the composition of the P(EDOT-HFIP-co-EDOT-C_n_) copolymer thin films coated on the plasma-treated silica substrates at a shallow depth. As shown in Figure 3a–c, the EDOT-C_n_ composition on the surface of all the thin films prepared by spin coating the copolymers synthesized under an EDOT-C_n_ feed ratio of 0.1 onto the plasma-treated silica substrates, is much higher than that of the corresponding copolymers dip-coated onto the pristine silica substrates. Additionally, the deviation of their surface compositions from that of the copolymer composition increases dramatically with the alkyl chain length (Figure 3c). As shown in Figure 3d–f, the EDOT-C_6_ surface composition of all the P(EDOT-HFIP-co-EDOT-C_6_) thin films spin-coated onto the plasma-treated silica substrates is significantly higher than that of the corresponding copolymers, regardless of the EDOT-C_6_ feed ratios. Furthermore, the deviation of their surface composition from the polymer composition also increases with the EDOT-C_6_ feed ratios (Figure 3f). All these results suggest the occurrence of phase segregation, where the alkyl-grafted EDOTs preferentially migrate to the surface, a process highly dependent on both the EDOT-C_n_ composition and the alkyl chain length. We attribute its underlying mechanistic cause to the strong interaction between the HFIP groups in the copolymers and the polar functionalities activated by the oxygen plasma on the surface. This interaction drives the EDOT-HFIP-rich segments toward the surface while simultaneously repelling the EDOT-C_n_-rich segments. Additionally, the more extended alkyl side groups likely enhance this phase segregation by weakening their interactions with the surface due to their increased nonpolarity and by improving the polymer mobility, as longer alkyl chains contribute a more significant mass fraction to the copolymers. Similarly, a higher EDOT-C_n_ composition likely promotes this phase segregation, as an increased number of alkyl chains enhances polymer mobility and facilitates segregation.

This surface-driven phase segregation of the copolymers of the EDOTs bearing groups with very different polarities, evidenced by the significantly increased EDOT-C_n_ composition on the surface, is likely a critical driving force for the nano-assembly of the copolymers. The consistent correlation between the phase segregation and nano-assembly dependence on the EDOT-C_n_ composition and the alkyl chain length strongly supports this consideration.

### 3.3. Specific Sensing and Water Resistance of P(EDOT-HFIP-Co-EDOT-C_n_) Sensors

We then similarly spin-coated these P(EDOT-HFIP-co-EDOT-C_6_) thin films onto the interdigitated electrodes, which were pretreated with oxygen plasma, to fabricate the organophosphate chemiresistive sensors (Figure S1). These sensors detect organophosphates through the dedoping of EDOT copolymers, driven by the specific interaction between the organophosphates and HFIP groups, which initially doped the EDOT copolymers [23]. The sensor response value reflects the sensor’s sensitivity, which was obtained by calculating the resistance change rate before and after the sensor came into contact with the targeted organophosphate molecules.

**Figure 4:**
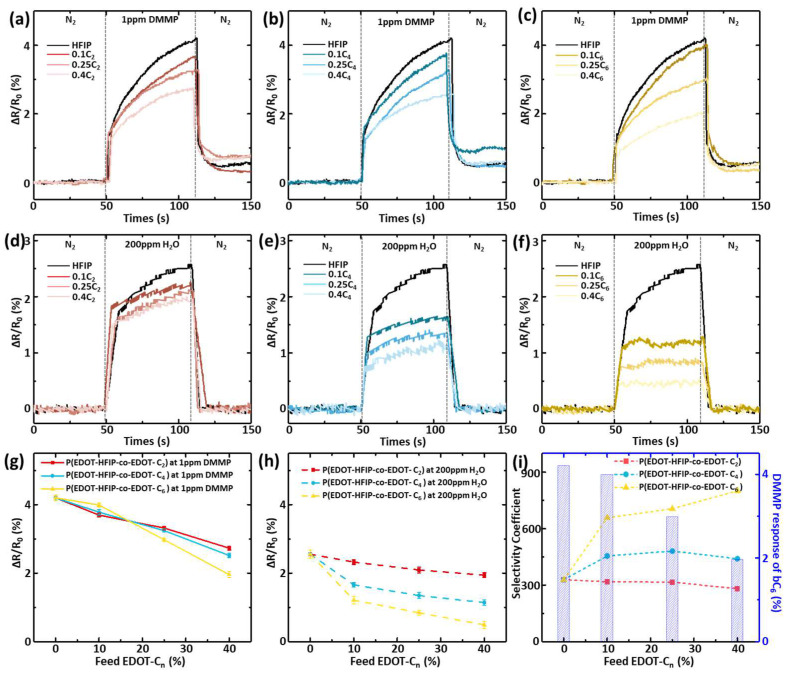
(**a**–**c**) Sensing curves of the chemiresistive sensors with varying P(EDOT-HFIP-co-EDOT-Cn) copolymers, exposed to 1 ppm DMMP vapor for 60 s. (**d**–**f**) Sensing curves of the chemiresistive sensors with varying P(EDOT-HFIP-co-EDOT-Cn) copolymers, exposed to 200 ppm water vapor for 60 s. (**g**) Comparison of the response values for DMMP (1 ppm, 1 min) of the above sensors. (**h**) Comparison of the response values for water (200 ppm, 1 min) of the above sensors. (**i**) Selectivity coefficient for DMMP/water (1 ppm, 1 min) (**left**) in the above sensors and the response values for DMMP (1 ppm, 1 min) (**right**) of the sensors with P(EDOT-HFIP-co-EDOT-C6).

As our primary concern was whether the sensing material’s ability to resist water vapor interference aligned with the original molecular design intent, we first evaluated the water resistance of these chemiresistive sensors by focusing on the effects of the copolymer compositions and the alkyl group lengths. Since the sensor’s response to water vapor was much smaller than its response to DMMP, we set the water vapor concentration to a very high value of 200 ppm to enhance the accuracy of the evaluation. During the measurement, the chemiresistive sensors were continuously subjected to a 50 s nitrogen (N_2_) gas purge, followed by exposure to 200 ppm of water vapor for 60 s to induce a resistive response, and then purged again with N_2_. The real-time resistance change was monitored for each P(EDOT-HFIP-co-EDOT-C_n_) sensor, as shown in Figure 4a–c, with the PEDOT-HFIP one as the control. The resistance change rates of the various polymers over 60 s are summarized in Figure 4g as a function of the EDOT-C_n_ feed ratios. As expected, the resistive response of the P(EDOT-HFIP-co-EDOT-C_2_) sensor decreased linearly with an increase in the EDOT-C_2_ feed ratio. However, the responses of the P(EDOT-HFIP-co-EDOT-C_4_) and P(EDOT-HFIP-co-EDOT-C_6_) sensors differed in their dependence on the EDOT-C_n_ feed ratio, with a significant drop at a 0.1 EDOT-C_n_ feed ratio, followed by a gradual decrease in the response as the EDOT-C_n_ feed ratio increased further. In detail, the water vapor responses of the P(EDOT-HFIP-co-EDOT-C_2_), P(EDOT-HFIP-co-EDOT-C_4_), and P(EDOT-HFIP-co-EDOT-C_6_) sensors decreased by 7.0%, 33.6%, and 51.5%, respectively. Notably, the P(EDOT-HFIP-co-EDOT-C_6_) sensor exhibited an impressive 51.5% decrease in water vapor response with only ~10% EDOT-C_6_. We are keen to delve into the underlying reasons for this phenomenon, and a detailed analysis will be presented later.

We subsequently studied the resistance response of the chemiresistive sensors integrated with these P(EDOT-HFIP-co-EDOT-C_n_) copolymers to 1 ppm DMMP, a common organophosphate compound used to evaluate organophosphate sensors (Figure 4d–f). The maximum resistance changes in these P(EDOT-HFIP-co-EDOT-C_n_) sensors are summarized in Figure 4h. Unfortunately, the results indicate that incorporating EDOT-C_n_ compromised sensor sensitivity, as the resistive response of the P(EDOT-HFIP-co-EDOT-C_n_) sensors decreased with the EDOT-C_n_ feed ratio. This result suggests that the enhancement of the water resistance of a chemiresistive sensor comes at the expense of sensitivity, which may significantly compromise the sensor’s potential for use in real-world applications.

We attribute the decrease in sensitivity to the incorporation of the copolymer monomers, which reduced the number of HFIP groups in the thin film, thereby weakening the resistive response. Interestingly, the sensor integrated with the P(EDOT-HFIP-co-EDOT-C_6_) synthesized under a 0.1 EDOT-C_6_ feed ratio showed only a minimal reduction (~5%) in its sensitivity, contrasting sharply with the sensors using the P(EDOT-HFIP-co-EDOT-C_2_) and P(EDOT-HFIP-co-EDOT-C_4_) with similar compositions, which showed more significant declines (≥10%). Based on the SEM images in Figure 2c, we attribute the nearly unchanged sensitivity of the chemiresistive sensor integrated with P(EDOT-HFIP-co-EDOT-C_6_) at a low EDOT-C_6_ composition to the increased specific surface area, which results from the higher density of the nanorods on the surface. Therefore, this P(EDOT-HFIP-co-EDOT-C_6_) sensor would likely allow us to fabricate a highly sensitive but water-resistant chemiresistive sensor.

Based on the resistive response of these copolymer sensors to organophosphate and water vapor, we calculated the selectivity coefficient (SC), defined as the ratio of the response to DMMP to that of water vapor at the same concentration (Equation (1)), to evaluate the sensing selectivity of the chemiresistive sensor, as shown in Figure 4i. It was observed that the P(EDOT-HFIP-co-EDOT-C_2_) sensor showed no improvement in the sensing selectivity compared to the PEDOT-HFIP. In contrast, the P(EDOT-HFIP-co-EDOT-C_4_) and P(EDOT-HFIP-co-EDOT-C_6_) sensors presented with a much improved sensing selectivity. Notably, all three P(EDOT-HFIP-co-EDOT-C_6_) sensors exhibited a sensing selectivity more than twice that of the PEDOT-HFIP, making them attractive candidates for selective organophosphate sensors for real-world sensing applications. Among them, the chemiresistive sensor integrated with P(EDOT-HFIP-co-EDOT-C_6_) synthesized at an EDOT-C_6_ feed ratio of 0.1 appears the most promising, as it retained nearly all of its original sensitivity while achieving a high selectivity of 658, comparable to that of the sensors using P(EDOT-HFIP-co-EDOT-C_6_) synthesized at higher EDOT-C_6_ feed ratios (706 and 801 for 0.25 and 0.4, respectively).

As described above, this P(EDOT-HFIP-co-EDOT-C_6_) sensor demonstrated exceptional sensing selectivity while retaining highly specific sensitivity, presenting a viable strategy for simultaneously optimizing these two typically competing characteristics in chemiresistive sensors. It was entirely unexpected that incorporating just 10% of the EDOT-C_6_ monomer into the copolymer would yield an a chemiresistive sensor with an organophosphate sensitivity 657 times higher than its sensitivity to water vapor. Considering the pronounced surface-driven phase segregation observed in the P(EDOT-HFIP-co-EDOT-C_6_) thin film, we attribute the significantly enhanced sensing selectivity to the substantial migration of the EDOT-C_6_-rich segments to the film surface. Indeed, the excellent agreement between the water resistance of these chemiresistive sensors (Figure 4h) and the EDOT-C_n_ enrichment of the film surface (Figure 3c,f) well support this explanation. Conversely, for the chemiresistive sensor P(EDOT-HFIP-co-EDOT-C_6_) with 10% EDOT-C_6_, the surface-driven phase segregation concentrated the EDOT-HFIP-rich segments below the surface, thereby preserving the original high sensitivity toward organophosphate. In this way, the surface-driven phase segregation simultaneously enhanced water resistance significantly and restored the specific sensitivity compromised by the incorporation of 10% EDOT-C_6_, resulting in a chemiresistive sensor with an organophosphate sensitivity 657 times greater than its sensitivity to water vapor.

### 3.4. Shelf Storage Stability of P(EDOT-HFIP-Co-EDOT-C_n_) Sensors

**Figure 5:**
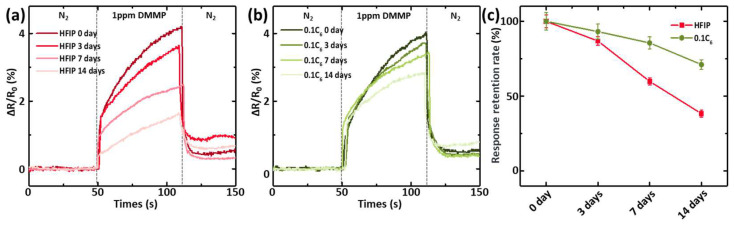
(**a**) Resistive responses of the chemiresistive sensors integrated with P(EDOT-HFIP-co-EDOT-C6) containing 10% EDOT-C6 to 1 ppm DMMP vapor, after being exposed directly to the air for 0, 3, 7, and 14 days. (**b**) Resistive responses of the chemiresistive sensors integrated with P(EDOT-HFIP) to 1 ppm DMMP vapor after exposure to the air for 0, 3, 7, and 14 days. (**c**) Dependences of the normalized resistive responses with the storage time for the chemiresistive sensor integrated with P(EDOT-HFIP-co-EDOT-C6) containing 10% EDOT-C6 and the PEDOT-HFIP sensor.

Chemiresistive sensors typically feature high sensitivity and a low detection limit, making storage in a dry inert gas atmosphere generally necessary. Storing these chemiresistive sensors on a shelf without specialized protection remains very challenging, particularly for those with a ppb detection limit [23]. To evaluate the impact of the surface-driven phase-segregation strategy on the shelf storage stability of these chemical sensors, we exposed them directly to the air for 14 days in our electronic device fabrication cleanroom (class 10,000), with the temperature and humidity of the environment maintained at 20–25 °C and 50–70%, respectively. During the storage period, the response of the chemiresistive sensor integrated with P(EDOT-HFIP-co-EDOT-C_6_) containing 10% EDOT-C_6_ was monitored, with the PEDOT-HFIP sensor as the control. As illustrated in Figure 5a–c, the response of the PEDOT-HFIP sensor to 1 ppm DMMP experienced a dramatic degradation of 61.7% over the 14 days of storage. In contrast, the P(EDOT-HFIP-co-EDOT-C_6_) sensor demonstrated a significantly improved shelf storage stability, with only a 28.9% reduction in sensitivity after the same 14-day shelf storage period. This significant enhancement in shelf storage stability can be attributed to the improved water resistance of the P(EDOT-HFIP-co-EDOT-C_6_) sensor. Additionally, the presence of organic solvent molecules in the cleanroom atmosphere, even at potentially low concentrations, may have introduced another challenge for the sensor, contributing to its degradation. We plan to investigate and address this issue in future studies.

## 4. Conclusions

In this study, we electro-copolymerized EDOT-HFIP with alkyl-grafted EDOT comonomers to engineer a series of water-resistant, yet organophosphate-sensitive, conducting polymers for chemiresistive sensors. The composition and alkyl group lengths of the alkyl-grafted EDOT comonomers were precisely modulated to improve the water resistance of the chemiresistive sensors while retaining the original organophosphate sensitivity of the PEDOT-HFIP sensor. However, improving the water resistance of the copolymer-integrated chemiresistive sensor by increasing the alkyl length and alkyl-bearing EDOT composition came at the unacceptable cost of compromising its organophosphate sensitivity. To solve the conflict between water resistance and organophosphate sensitivity, we treated the silica substrates using oxygen plasma to induce phase segregation in the thin films during spin coating, thus enriching the alkyl chains on the surface while concentrating the HFIP groups beneath it. This surface-driven phase-segregation strategy efficiently decoupled and optimized these competing sensor characteristics for the sensor integrated with the EDOT-HFIP copolymer containing 10% hexyl-grafted EDOT. This EODT-HFIP copolymer sensor demonstrated an organophosphate (DMMP) resistive response 657 times higher than that to water vapor, and more than two times that of the PEDOT-HFIP sensor, while preserving the original specific sensitivity of the PEDOT-HFIP sensor. Finally, it exhibited a much improved shelf storage stability compared to that of the PEDOT-HFIP sensor, retaining 71.1% sensitivity even after 14 days of direct air exposure without any specialized protection, while the PEDOT-HFIP sensor retained only 38.3%. We envision this surface-driven phase-segregation strategy will offer a promising solution to the longstanding challenge of air moisture interference in highly sensitive polymer sensors.

## Data Availability

The original contributions presented in this study are included in the article/Supplementary Material. Further inquiries can be directed to the corresponding author.

## Supplementary Materials

The following supporting information can be downloaded at: https://www.mdpi.com/article/10.3390/polym17070979/s1, Figure S1: Schematic diagram of organophosphorus sensing; Figure S2: Design drawing of the dedicated interdigitated electrode (IDE); Figure S3: Processing process flow chart for the IDE (1. silicon substrate, 2. silicon dioxide insulating layer, 3. photoresist, 4. chromium adhesive layer, and 5. gold conductive layer); Figure S4: Photograph of the IDE; Figure S5: I–V curves registered during the electrodepositions; Figure S6: SEM view of P(EDOT-HFIP-co-EDOT-C_6_) copolymer synthesized under 40% EDOT-C_6_ feed ratio and spin-coated on untreated silica substrate.

## References

[1] Vallero D.A. (2014). Fundamentals of Air Pollution—Fifth Edition.

[2] Yang L., Qin M., Zhang G., Yang J., Yang J., Zhao J. (2023). Progress of sensitive materials in chemiresistive sensors for detecting chemical warfare agent simulants: A review. Rev. Anal. Chem..

[3] Najafi P., Ghaemi A. (2024). Chemiresistor gas sensors: Design, Challenges, and Strategies: A comprehensive review. Chem. Eng. J..

[4] Patil L.A., Deo V.V., Shinde M.D., Bari A.R., Kaushik M.P. (2011). Sensing of 2-chloroethyl ethyl sulfide (2-CEES)—A CWA simulant—Using pure and platinum doped nanostructured CdSnO3 thin films prepared from ultrasonic spray pyrolysis technique. Sens. Actuators B Chem..

[5] Yang Z., Zhang Y., Zhao L., Fei T., Liu S., Zhang T. (2022). The synergistic effects of oxygen vacancy engineering and surface gold decoration on commercial SnO2 for ppb-level DMMP sensing. J. Colloid Interface Sci..

[6] Dai Z., Duan G., Cheng Z., Xu L., Li T., Liu G., Zhang H., Li Y., Cai W. (2015). Janus gas: Reversible redox transition of Sarin enables its selective detection by an ethanol modified nanoporous SnO2 chemiresistor. Chem. Commun..

[7] Fan Y., Li K., Ren X., Yan W., Zhu C., Zhao Y., Zeng W., Chen Z., Wang S. (2021). A highly selective gas sensor based on the WO3/WS2 van der Waals heterojunction for the 2-chloroethyl ethyl sulfide (2-CEES) sensing application. J. Mater. Chem. C Mater. Opt. Electron. Devices.

[8] Yoo R., Cho S., Song M.-J., Lee W. (2015). Highly sensitive gas sensor based on Al-doped ZnO nanoparticles for detection of dimethyl methylphosphonate as a chemical warfare agent simulant. Sens. Actuators B Chem..

[9] Jiang W., Jiang M., Wang T., Chen X., Zeng M., Yang J., Zhou Z., Hu N., Su Y., Yang Z. (2021). Room temperature DMMP gas sensing based on cobalt phthalocyanine derivative/graphene quantum dot hybrid materials. RSC Adv..

[10] Wang Y., Yang M., Liu W., Dong L., Chen D., Peng C. (2019). Gas sensors based on assembled porous graphene multilayer frameworks for DMMP detection. J. Mater. Chem. C Mater. Opt. Electron. Devices.

[11] Hu N., Wang Y., Chai J., Gao R., Yang Z., Kong E.S.-W., Zhang Y. (2012). Gas sensor based on p-phenylenediamine reduced graphene oxide. Sens. Actuators B Chem..

[12] Kim Y.-T., Lee S., Park S., Lee C.Y. (2019). Graphene chemiresistors modified with functionalized triphenylene for highly sensitive and selective detection of dimethyl methylphosphonate. RSC Adv..

[13] Wiederoder M.S., Nallon E.C., Weiss M., McGraw S.K., Schnee V.P., Bright C.J., Polcha M.P., Paffenroth R., Uzarski J.R. (2017). Graphene Nanoplatelet-Polymer Chemiresistive Sensor Arrays for the Detection and Discrimination of Chemical Warfare Agent Simulants. ACS Sens..

[14] Yu H., Han H., Jang J., Cho S. (2019). Fabrication and Optimization of Conductive Paper Based on Screen-Printed Polyaniline/Graphene Patterns for Nerve Agent Detection. ACS Omega.

[15] Saetia K., Schnorr J.M., Mannarino M.M., Kim S.Y., Rutledge G.C., Swager T.M., Hammond P.T. (2014). Spray-Layer-by-Layer Carbon Nanotube/Electrospun Fiber Electrodes for Flexible Chemiresistive Sensor Applications. Adv. Funct. Mater..

[16] Liu Y., Chen C.-L., Zhang Y., Sonkusale S.R., Wang M.L., Dokmeci M.R. (2013). SWNT based nanosensors for wireless detection of explosives and chemical warfare agents. IEEE Sens. J..

[17] Kong L., Wang J., Luo T., Meng F., Chen X., Li M., Liu J. (2010). Novel pyrenehexafluoroisopropanol derivative-decorated single-walled carbon nanotubes for detection of nerve agents by strong hydrogen-bonding interaction. Analyst.

[18] Wang F., Gu H., Swager T.M. (2008). Carbon nanotube/polythiophene chemiresistive sensors for chemical warfare agents. J. Am. Chem. Soc..

[19] Yoo R., Kim J., Song M.-J., Lee W., Noh J.S. (2015). Nano-composite sensors composed of single-walled carbon nanotubes and polyaniline for the detection of a nerve agent simulant gas. Sens. Actuators B Chem..

[20] Wang Z., Yu J., Gui R., Jin H., Xia Y. (2016). Carbon nanomaterials-based electrochemical aptasensors. Biosens Bioelectron..

[21] Wong Y.C., Ang B.C., Haseeb A.S.M.A., Baharuddin A.A., Wong Y.H. (2020). Review—Conducting polymers as chemiresistive gas sensing materials: A review. J. Electrochem. Soc..

[22] Bai H., Shi G. (2007). Gas sensors based on conducting polymers. Sensors.

[23] Luo B., Weng J., Geng Z., Pan Q., Pei X., He Y., Chen C., Zhang H., Wei R., Yuan Y. (2022). Solution-processed wafer-scale nanoassembly of conducting polymers enables selective ultratrace nerve agent detection at low power. Nano Res..

[24] Grate J.W., Klusty M., Barger W.R., Snow W. (1990). Role of selective sorption in chemiresistor sensors for organophosphorus detection. Anal. Chem..

[25] Chahal M.K., Sumita M., Labuta J., Payne D.T., Hill J.P., Yamauchi Y., Nakanishi T., Tanaka T., Kataura H., Koga K. (2023). Selective Detection of Toxic C1 Chemicals Using a Hydroxylamine-Based Chemiresistive Sensor Array. ACS Sens..

[26] Tiggemann L., Ballen S.C., Bocalon C.M., Graboski A.M., Manzoli A., Steffens J., Valduga E., Steffens C. (2017). Electronic nose system based on polyaniline films sensor array with different dopants for discrimination of artificial aromas. Innov. Food Sci. Emerg..

[27] Rath R.J., Farajikhah S., Oveissi F., Dehghani F., Naficy S. (2022). Chemiresistive Sensor Arrays for Gas/Volatile Organic Compounds Monitoring: A Review. Adv. Energy Mater..

[28] Lin H.A., Luo S.C., Zhu B., Chen C., Yamashita Y., Yu H.H. (2013). Molecular or Nanoscale Structures? The Deciding Factor of Surface Properties on Functionalized Poly(3,4-ethylenedioxythiophene) Nanorod Arrays. Adv. Funct. Mater..

