# Supplementary material for: Surface-Driven Phase Segregation in Conducting Polymer Thin Films Enables High Selectivity and Storage Stability of Chemiresistive Sensors in Humid Air
Source: Polymers (Basel). 2025 Apr 3;17(7):979. doi: 10.3390/polym17070979 (PMC11991412; doi:10.3390/polym17070979)
Supplement: Supplementary file 1 [file polymers-17-00979-s001.zip › polymers-3550611-supplementary.pdf]

Supplementary Materials: Surface-Driven Phase Segregation in Conducting Polymer Thin Films Enables High Selectivity and Storage Stability of Chemiresistive Sensors in Humid Air

Jianan Weng [1,2,3], Wei Wu [1,2,3], Minghao Qian [1,2,3], Jiarui Zhang [1,2,3], Shuhua Zhang [1,2,3], Zhi Geng [1,2,3] and Bo Zhu [1,2,3,*]

1 School of Materials Science and Engineering, 99 Shangda Road, Baoshan, Shanghai 200444, China
2 Shanghai Engineering Research Center of Organ Repair, 99 Shangda Road, Baoshan, Shanghai 200444, China
3 Joint International Research Laboratory of Biomaterials and Biotechnology in Organ Repair (Ministry of Education), 99 Shangda Road, Baoshan, Shanghai 200444, China
* Correspondence: bozhu@shu.edu.cn

1. Schematic diagram of organophosphorus sensing

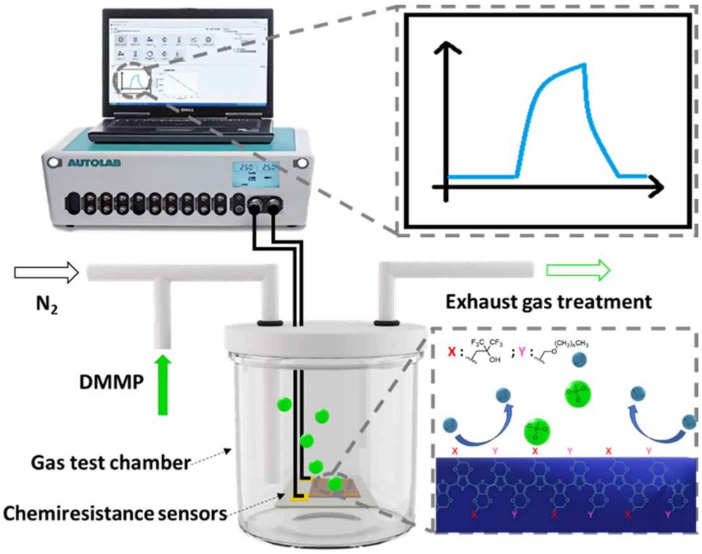

Figure S1. Schematic diagram of organophosphorus sensing.

2. Design and fabrication of the specially interdigitated electrode (IDE)

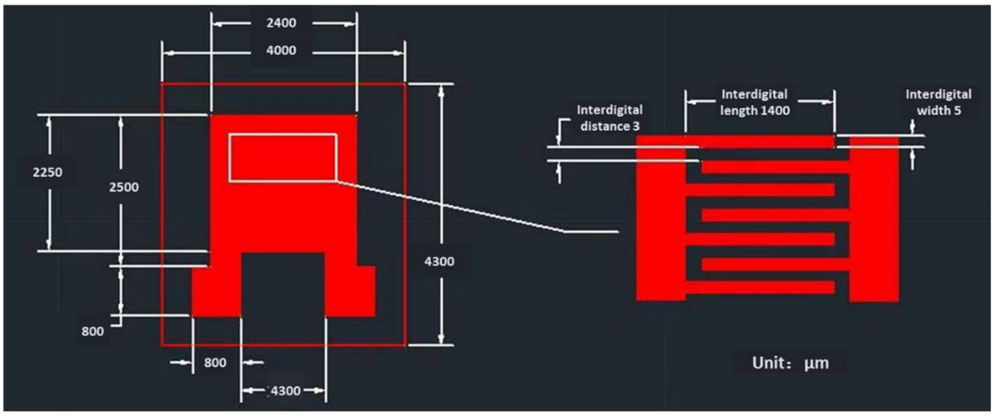

Figure S2. Design drawing of the dedicated interdigitated electrode (IDE).

The IDE has the functions of signal amplification and power consumption reduction. By increasing the number of fork–finger pairs to increase the equivalent area between the two electrodes, the change in resistance caused by gas adsorption on the sensitive material is amplified. At the same time, because the fork–finger pairs are connected in parallel with each other, the overall resistance of the device can be significantly reduced, thus reducing the power consumption. In the IDE design, the number of fingers was maximized to enhance the signal amplification, while the power consumption was minimized by reducing the electrode size and channel spacing in consideration of the processing technology limitations. The final IDE was designed as shown in Figure S2, with a fork–finger length of 1400μm, fork–finger width of 5μm, fork–finger spacing of 3μm, and 140 fork–finger pairs.

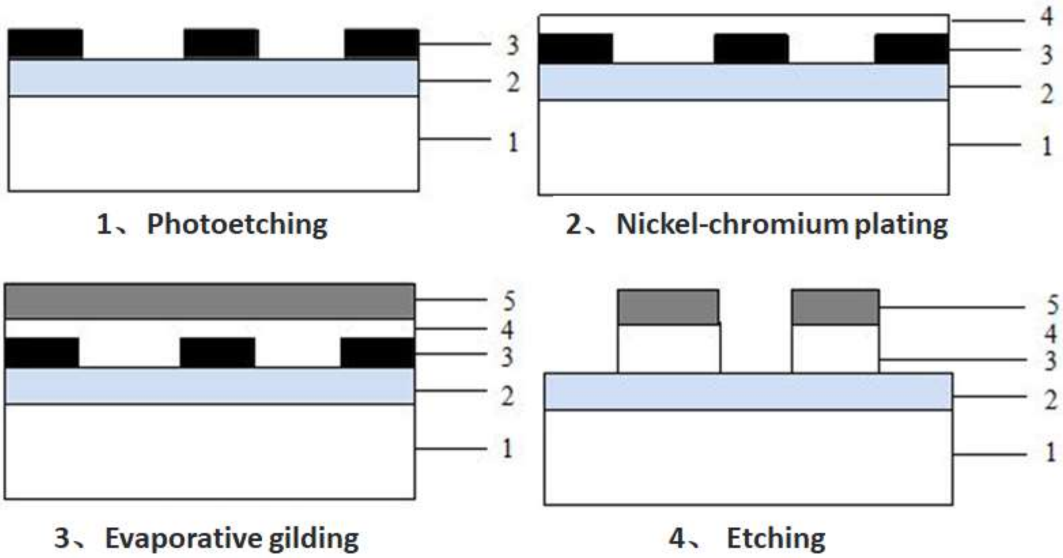

Figure S3. Processing process flow chart for the IDE (1. silicon substrate, 2. silicon dioxide insulating layer, 3. photoresist, 4. Nichel-chromium adhesive layer, and 5. gold conductive layer).

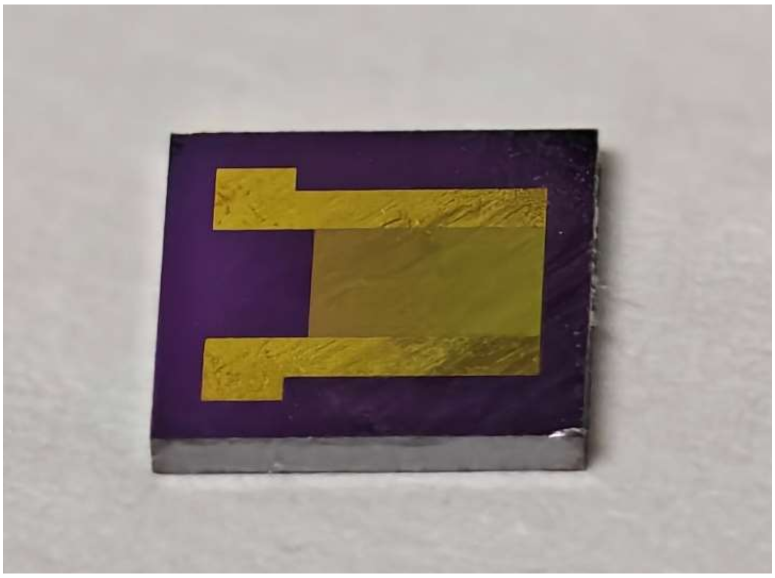

Figure S4. Photograph of the IDE

3. Electropolymerization of P(EDOT-HFIP-co-EDOT-C_n)s

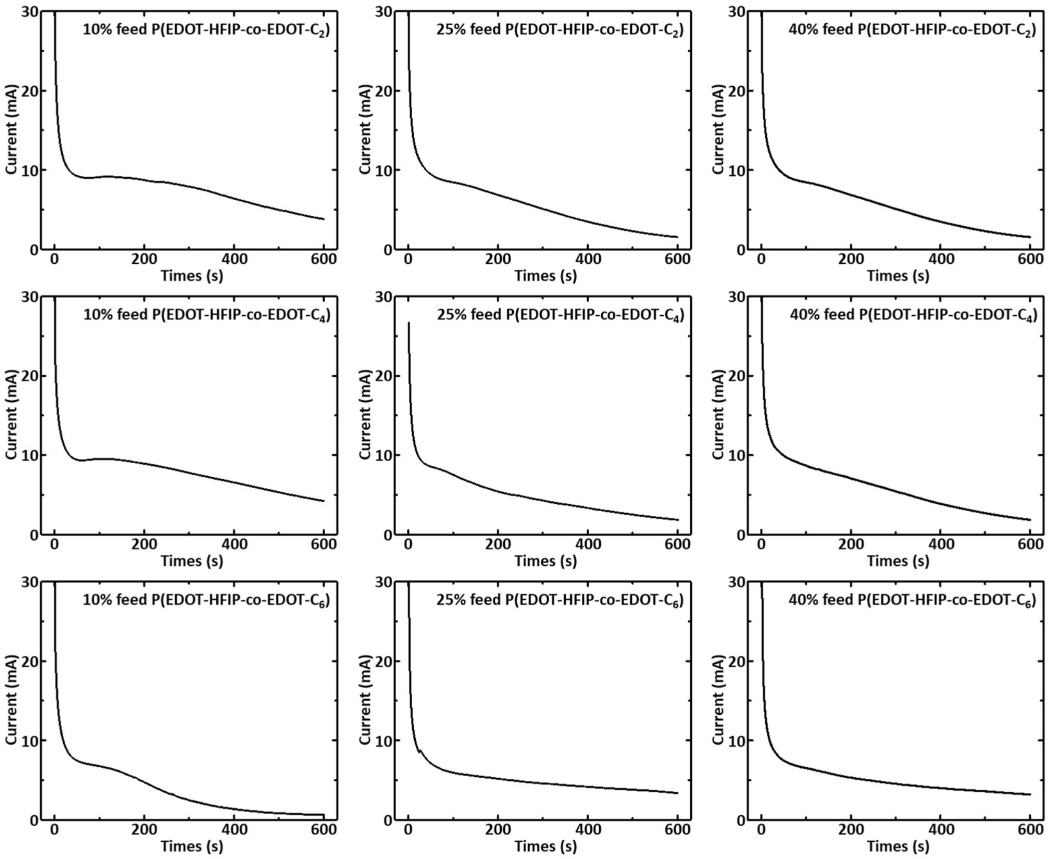

Figure S5. I–V curves recorded during the electrodeposition of EDOT-HFIP mixed with EDOT-C_2, EDOT-C_4, or EDOT-C_6 (at varying molar ratios of EDOT-Cn of 0, 0.1, 0.25, and 0.4) onto a Pt mesh substrate under a constant potential of 1.13 V in an aqueous electrolyte using a Ag/AgCl reference electrode.

4. SEM view of copolymer films spin-coated onto untreated silica substrate

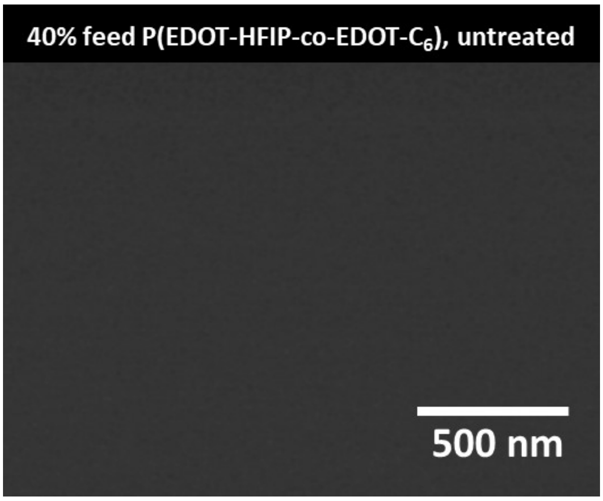

Figure S6. SEM view of P(EDOT-HFIP-co-EDOT-C_6) copolymer synthesized under 40% EDOT-C_6 feed ratio and spin-coated onto an untreated silica substrate.
